# EDITORIAL

**DOI:** 10.1017/S2633903X2000001X

**Published:** 2021-01-11

**Authors:** Jean-Christophe Olivo-Marin

**Affiliations:** Institut Pasteur, BioImage Analysis Unit, Paris, France

It is a real pleasure to announce the launch of *Biological Imaging*, a new open access journal published by Cambridge University Press, dedicated to the blooming field of quantitative and computational imaging in life sciences.

Starting a new journal is a collective adventure and I am particularly delighted to be working with the recognised experts in the Executive Editorial Board and Associate Editorial Board (https://www.cambridge.org/core/journals/biological-imaging/information/editorial-board) who have accepted to tackle the challenge of launching and editing *Biological Imaging.* They all are outstanding scientists who bring a large and diverse palette of expertise and views covering the different aspects of biological imaging, and will be commissioning and handling papers for the journal. I should also mention the fantastic people at Cambridge University Press (https://www.cambridge.org/core/what-we-publish/open-access) who have been, and will be counselling and assisting us in reaching and maintaining our objectives.

Quantitative and computational bioimaging is a recent interdisciplinary scientific area that brings together the power of physical, computer, and mathematical sciences to the biological field, to image and extract comprehensive information from large data sets and quantitatively characterize biological experiments. Indeed, studying the biology of natural systems, such as cells, whole organisms, or reconstituted systems like organs-on-chips, increasingly relies on imaging to visualize, measure, model these systems, and, eventually, to uncover their inner workings. This is done through the combined use of hardware and software set-ups, methods and algorithms developed in inter-connected fields such as optics, biophysics, signal and image processing, computer vision, computational modelling and computer science, without forgetting the increasing role of machine learning.

In spite, or because of the large span of disciplines involved, and probably also because of its still underestimated role and recognition, there are currently only few journals that specifically cater for bioimaging, and none that covers altogether the central contributions of image processing and analysis, data mining and analysis, mathematical modelling and machine learning, topics that are currently scattered across a range of journals, and more often than not, relegated to Supplementary Information and data.

*Biological Imaging* differentiates itself from existing journals in many aspects. First, it provides a single venue and open access interdisciplinary forum for important bioimaging research. Second, it aims to provide a home for extensive and thorough methods papers and welcomes submissions ranging the spectrum from new experimental protocols, combined hardware-software computational imaging devices, to core algorithms and software methods and tools. Third, it publishes original research articles, reviews and short reports on techniques and methods that use quantitative and computational imaging at their core and that are prone to enable discoveries and advances in biology. Then, while promoting open science and data sharing, the journal strives at setting high publication standards in software quality and usability, as well as data collection, collation, and analysis.

We strongly believe that because bioimaging is a domain evolving at fast pace, the diffusion of emerging ideas and methods need be accelerated from the dry to the wet labs, and vice versa. We have the vision that *Biological Imaging*, through the quality of the papers and its fast and widespread publication policy, will help indeed to create cross fertilization across the spectrum of biological imaging research and applications and spur a variety of innovative and groundbreaking work taking root in the published articles.

Our hope is that you, the large community of scientists working in this exciting domain, will join us in building *Biological Imaging* as a first in-class resource and journal and we encourage you to submit your best manuscripts from now on. We also look forward to receiving your suggestions, comments and ideas for future developments.

